# Ferroelectric HfO_2_–ZrO_2_ Multilayers with Reduced Wake-Up

**DOI:** 10.1021/acsomega.4c10603

**Published:** 2025-03-28

**Authors:** Barnik Mandal, Adrian-Marie Philippe, Nathalie Valle, Emmanuel Defay, Torsten Granzow, Sebastjan Glinsek

**Affiliations:** † Smart Materials Unit, Luxembourg Institute of Science and Technology (LIST), 41 Rue de Brill, L-4422 Belvaux, Luxembourg; ‡ University of Luxembourg, 2 Av. de l’Universite, Esch-Belval, L-4365 Esch-sur-Alzette, Luxembourg; § Advanced Analyses and Support Unit, Luxembourg Institute of Science and Technology (LIST), 41 Rue de Brill, L-4422 Belvaux, Luxembourg

## Abstract

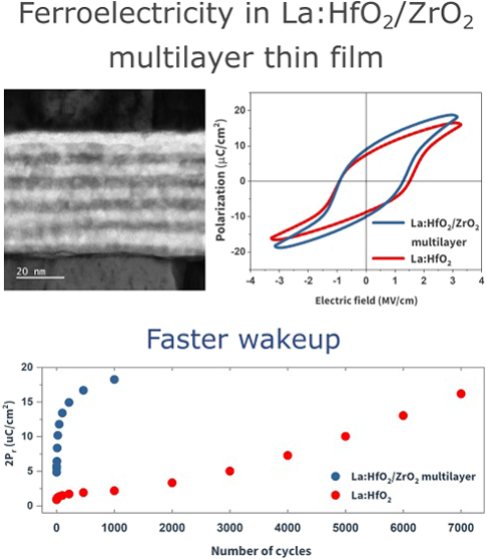

Since the discovery of ferroelectricity in HfO_2_ thin films, significant research has focused on Zr-doped HfO_2_ and solid-solution (Hf,Zr)O_2_ thin films. Functional properties can be further tuned via multilayering; however, this approach has not yet been fully explored in HfO_2_–ZrO_2_ films. This work demonstrates ferroelectricity in a 50 nm-thick, solution-processed HfO_2_–ZrO_2_ multilayer film, marking it as the thickest multilayer film to date exhibiting ferroelectric properties. The multilayer structure was confirmed through transmission electron microscopy (TEM) and energy-dispersive X-ray spectroscopy, with high-resolution TEM revealing grain continuity across multiple layers. This finding indicates that a polar phase in the originally paraelectric ZrO_2_ layer can be stabilized by the HfO_2_ layer. The film attains a remanent polarization of 9 μC cm^–2^ and exhibits an accelerated wake-up behavior, attributed to its higher breakdown strength, resulting from the incorporation of multiple interfaces. These results offer a faster wake-up mechanism for thick ferroelectric hafnia films.

## 1 Introduction

The discovery of ferroelectricity in doped HfO_2_ has garnered interest due to its compatibility with complementary metal-oxide-semiconductor (CMOS) technology.^1^ Given the similar chemical and physical properties of HfO_2_ and ZrO_2_, studies on ferroelectricity in ZrO_2_ have also been conducted.^2^ Both of these simple oxides are regarded as promising lead-free alternatives for nonvolatile memory and piezoelectric applications. In 2016, Fan et al. demonstrated the stabilization of the ferroelectric orthorhombic phase in a ZrO_2_ thin film produced by radio frequency magnetron sputtering, utilizing substrate-induced strain to promote the transition from the paraelectric tetragonal phase (t-phase) to the ferroelectric orthorhombic phase (o-phase).^3^ Starschich et al. reported thicker ZrO_2_ and Hf-doped ZrO_2_ ferroelectric films of 100 and 390 nm, respectively, using chemical solution deposition (CSD) with organometallic precursors.^2^ The evolution of ferroelectricity in solid solutions of HfO_2_ and ZrO_2_ (Hf_*x*_Zr_1–*x*_O_2_) has been intensively studied using various growth techniques, with reported properties ranging from ferroelectric- to antiferroelectric-like. The maximum remanent polarization *P*_r_ is observed typically around the composition Hf_0.5_Zr_0.5_O_2_, while the existence of a CMOS-compatible morphotropic phase boundary has been postulated for the composition Hf_0.3_Zr_0.7_O_2_.^4−6^

Several experimental investigations reported ferroic HfO_2_–ZrO_2_ multilayers.^7−11^ The main drive behind the majority of these studies has been the stabilization of the ferroelectric phase and enhancement of properties in sub-20 nm multilayer films through mechanical confinement of the layers,^7^ increased interface energy,^8^ and lattice distortion caused by grain coalescence during thermal annealing.^9^ Using density functional theory, Dutta et al. demonstrated that small-sized dopants like Si tend to form distinct layers in the orthorhombic ferroelectric polymorph, avoiding intermixing with Hf. Layering stabilizes the polar phase over nonpolar polymorphs, strongly suggesting the use of multilayer structures instead of solid solutions.^12^ Furthermore, Cheema et al. reported permittivity enhancement in ultrathin HfO_2_–ZrO_2_ superlattices with a mixed ferroelectric-antiferroelectric order, i.e., an effect not possible in conventional ferroelectrics.^11^ These results demonstrate the potential advantages of multilayers in polar fluorite oxides.

In this work, we examine the stabilization of ferroelectricity and the wake-up behavior in 50 nm multilayer La:HfO_2_–ZrO_2_ thin films, representing the thickest multilayer thin films studied to date. While there have been studies on thick Hf_0.5_Zr_0.5_O_2_ solid-solution films,^2,13^ they have not specifically focused on multilayer structures. We prepared pure ZrO_2_, La:HfO_2_, and multilayer La:HfO_2_–ZrO_2_ films using a CSD process described in our previous publication.^14^ La doping for the HfO_2_ layer was chosen based on density functional theory predictions, which suggested that La’s larger ionic radius and lower electronegativity promote stabilization of the ferroelectric orthorhombic *Pca*2_1_ phase.^15,16^ Experimental findings further validated the predictions, demonstrating a wide doping range and reduced polarization relaxation.^17^ For clarity in the following discussion, the thin-film samples of La:HfO_2_, ZrO_2_, and La:HfO_2_–ZrO_2_ multilayers are termed HO, ZO, and HZO, respectively. It is observed that although the pure ZrO_2_ film is paraelectric, the combination with the polar HfO_2_ layer induces ferroelectricity in the ZrO_2_ layer of the HZO film. High-resolution transmission electron microscopy (HRTEM) confirms that the polar phase extends through multiple layers. The HZO film exhibits ferroelectric properties with a remanent polarization (*P*_r_) of 9 μC cm^–2^, which is a minor improvement to the conventional HO films. We further show that the multilayered HZO films can sustain higher electric fields than the HO film alone, resulting in the significant reduction of wake-up cycles. The number of cycles required for polarization saturation in HZO decreased 10-fold (from 10,000 to 1000) compared to standard HO films.

## 2 Methods

### 2.1 Solution Preparation

Two 0.25 M precursor solutions were prepared, one containing La:HfO_2_ for the HO film and the other containing ZrO_2_ for the ZO film. Additionally, two solutions of La:HfO_2_ and ZrO_2_ with a concentration of 0.08 M were prepared for the multilayer HZO film. For 5% La-doped HfO_2_, Hf(IV)-acetylacetonate (Alfa Aesar, 97%) and La(III)-acetate hydrate (Sigma-Aldrich, 99.9%) were used as metal precursors, with La(III)-acetate hydrate being freeze-dried for 16 h to remove crystal water. For pure ZrO_2_, Zr(IV)-acetylacetonate (Sigma-Aldrich, 97%) was used. The powders were dissolved in propionic acid (Sigma-Aldrich, 99.5% purity) and then refluxed for 3 h at 150 °C with magnetic stirring in an Ar atmosphere using a modified Schlenk apparatus.

### 2.2 Film Preparation

**Figure 1:**
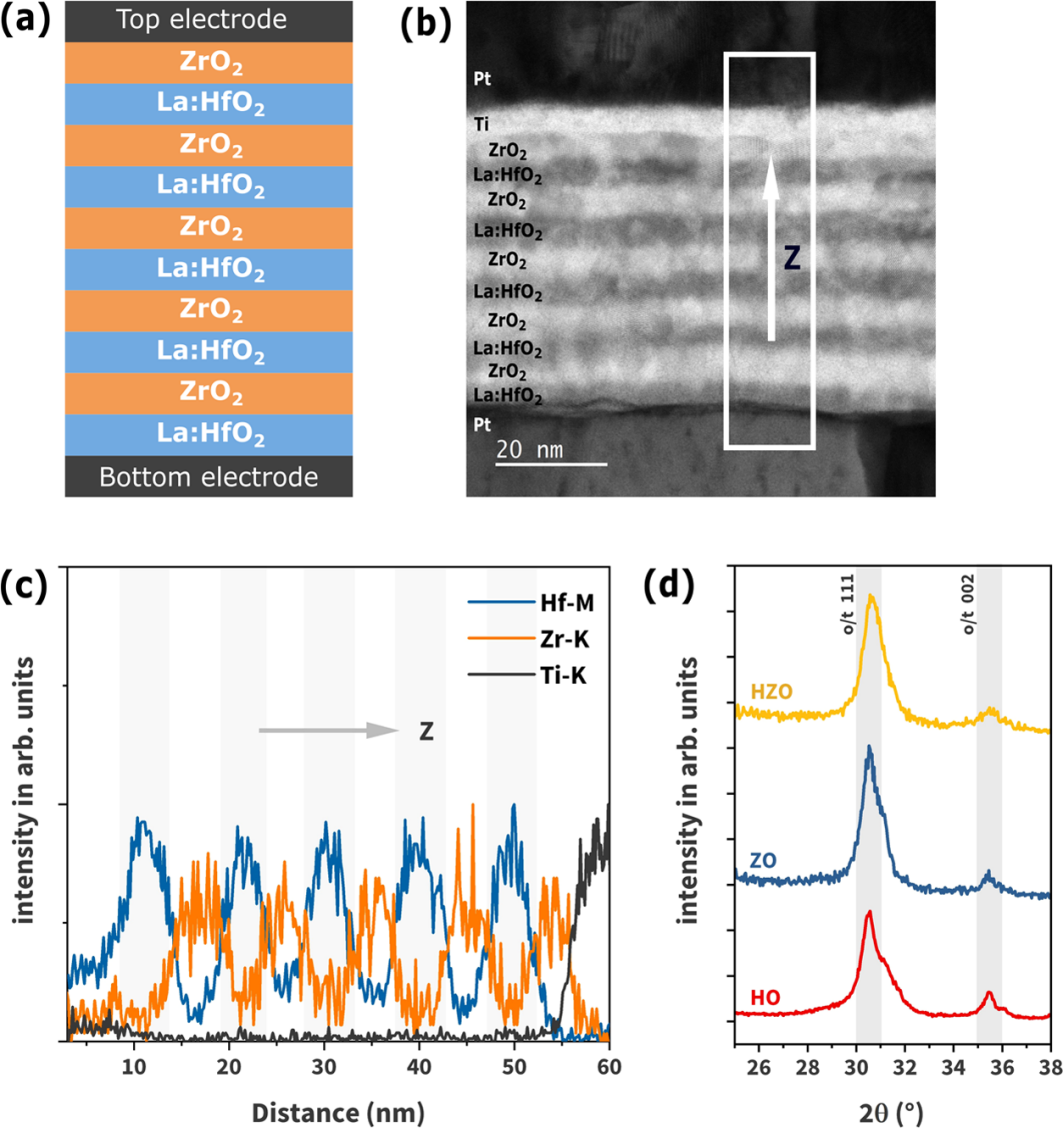
(a) Structural schematics and (b) bright-field STEM image of the HZO multilayer film with the Pt/Ti top electrode and the Pt bottom electrode. (c) STEM-energy dispersive X-ray spectroscopy (STEM-EDS) line profile of the HZO multilayer. *Z* is the direction of growth. (d) Grazing incidence X-ray diffraction (GIXRD) patterns of the HO film, ZO film, and HZO multilayer film. The main peaks are labeled with o/t (orthorhombic/tetragonal), which correspond to the reference patterns of *Pca*2_1_ and *P*4_2_/*nmc* phases, respectively.^18^

The solutions were spin-coated onto platinized Si substrates (Pt–Si, SINTEF) at 3000 rpm for 30 s. The platinum layer was strongly oriented in the (111) direction. After each spin-coating, a drying step was performed on a hot plate at 215 °C for 5 min. For the ZO and HO films, three 15 nm-thick amorphous layers were spin-coated from 0.25 M ZrO_2_ and 0.25 M La:HfO_2_ solutions, respectively. For the HZO sample, 10 5 nm-thick layers of La:HfO_2_ and ZrO_2_ were alternately deposited using 0.08 M La:HfO_2_ and 0.08 M ZrO_2_ solutions (see Figure 1a). The films were subsequently subjected to rapid thermal annealing (RTA) using an AS-Master 2000 (Annealsys) tool. In the RTA process, the chamber underwent a purging sequence by pumping down to a pressure below 4 mbar. Subsequently, the chamber was restored to atmospheric pressure while being filled with N_2_ and O_2_. N_2_ and O_2_ were pumped at flow rates of 1000 sccm each to establish an atmosphere in a 1:1 ratio. Following the chamber filling, the final crystallization step was initiated by reducing gas flow rates to 150:150 sccm for N_2_/O_2_. Films were then crystallized at 800 °C for 90 s with a 50 °C s^–1^ ramping rate. Circular top electrodes were evaporated on the film, each measuring 100 μm in diameter. These electrodes comprised two layers: first a 5 nm-thick Ti adhesion layer, followed by a 100 nm-thick Pt layer. The deposition was carried out using a Plassys evaporator under a pressure of 5 × 10^–8^ mbar. The electrodes were patterned by standard lithography and lift-off processes.

### 2.3 Characterization

A Bruker D8 Discover X-ray diffractometer with Cu Kα radiation (λ = 0.154 nm) was used for grazing incidence (GI) and θ–2θ X-ray diffraction (XRD). An incidence angle of 0.5° was used for GIXRD. The reference patterns for monoclinic, orthorhombic, and tetragonal HfO_2_ were taken from the powder diffraction files with the numbers 00-034-0104, 04-005-5597, and 04-003-2612, respectively.^18^ These same references were also applied to ZrO_2_, as they have very similar crystalline structures.

Ferroelectric measurements were performed using a TF Analyzer 2000 (aixACCT, Germany) instrument. Polarization versus electric field loops were acquired using a 3 kHz bipolar triangular signal, following the application of a desired number of rectangular wake-up cycles at 3 kHz. Two distinct sets of wake-up cycles were conducted on the HZO and HO films. The first set, referred to as procedure 1, consisted of a 1000-cycle wake-up at 15 V. The second set, referred to as procedure 2, involved 7000 cycles, performed as seven consecutive 1000-cycle wake-ups, with increasing voltages from 8 to 15 V.

The scanning transmission electron microscopy (STEM) analyses were performed on a JEOL JEM-F200 cold FEG microscope operating at an acceleration voltage of 200 kV. The TEM lamella was prepared following the “lift-out” method with a FEI Helios Nanolab 650 focused ion beam scanning electron microscope (FIB-SEM). X-ray energy-dispersive spectroscopy (EDS), using dual JEOL 100 mm^2^ silicon drift detectors, was performed in STEM mode allowing elemental mapping analysis. High-resolution TEM (HRTEM) imaging combined with fast Fourier transform (FFT) computation was performed to identify the crystalline phases observed throughout the thin film. The reference patterns for monoclinic, orthorhombic, and tetragonal HfO_2_ were taken from the powder diffraction files with the numbers 04-002-2772, 04-005-5597, and 04-027-1007, respectively.^18^

## 3 Results and Discussion

Figure 1a presents a structural schematic of the multilayer HZO film, which consists of ten 5 nm-thick layers of alternately deposited La:HfO_2_ and ZrO_2_, starting with La:HfO_2_ and ending with ZrO_2_. The bright-field STEM image shown in Figure 1b reveals the expected multilayered stack, indicated by the contrast variation between the La:HfO_2_ and ZrO_2_ layers, with two Pt-based electrodes enclosing the stack. The EDS spectrum (see the Supporting Information) to generate the line profile shown in Figure 1c was extracted from the area outlined by the white box shown in Figure 1b. The oscillating behavior of the Hf M and Zr K EDS line profiles shown in Figure 1c, along with EDS cross-sectional mapping (Supporting Information), further validates the multilayered structure of the film, with the Ti K line indicating where the film terminates. The grazing incident X-ray diffraction (GIXRD) patterns of the HO, ZO, and HZO films are illustrated in Figure 1d. The patterns show the characteristic features typically observed in HfO_2_ and ZrO_2_ thin films, i.e., a pronounced peak at 30.6° and a weaker peak at 35.5°, indicative of the orthorhombic polar planes (111) and (002), respectively. However, note that the structural similarities between the polar orthorhombic and nonpolar tetragonal phases lead to a significant overlap of their reflections, making it challenging to distinguish them in the laboratory XRD experiment. Additionally, a broad shoulder peak between ∼31° and 31.5° is also present, which may correspond to a monoclinic phase (*P*2_1_/*c*).

**Figure 2:**
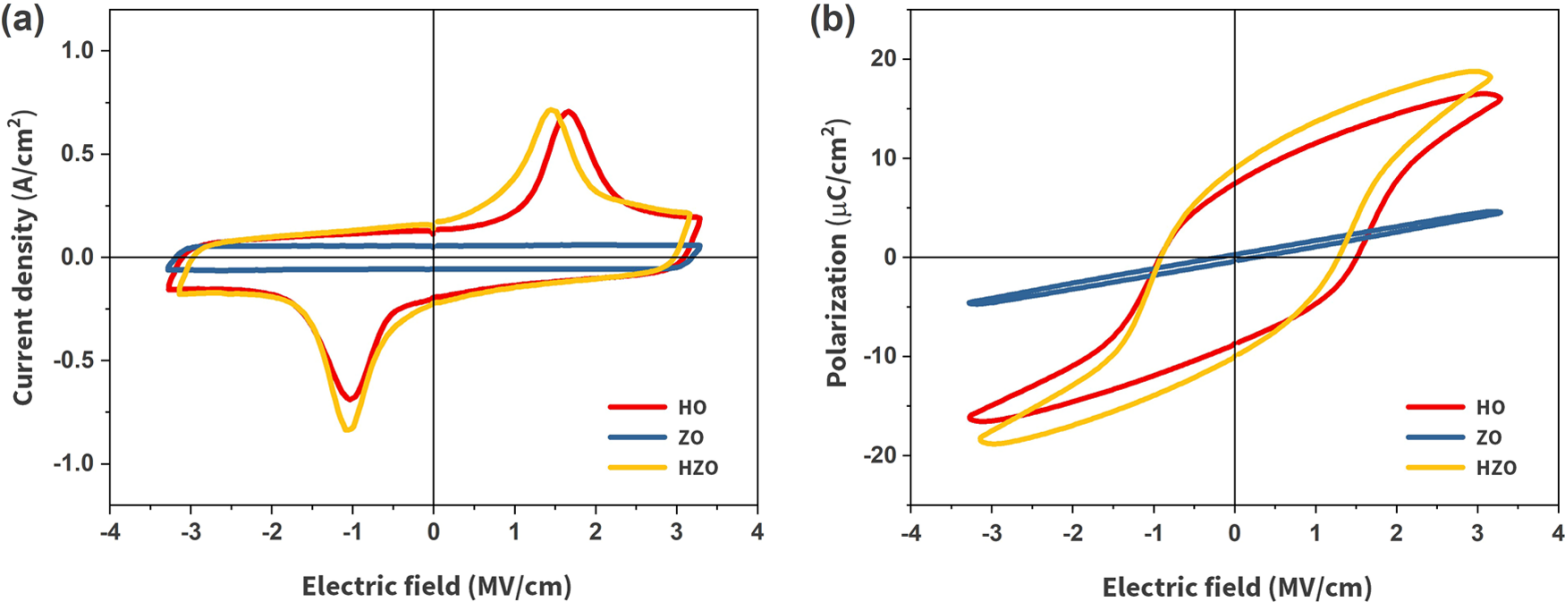
(a) Current density versus electric field loops and (b) polarization versus electric field loops of the three films.

The current density–electric field and polarization–electric field hysteresis loops after wake-up cycling are shown in Figure 2. The ZO film does not show any switching current and is therefore paraelectric. The HO and multilayered HZO films exhibit prominent switching current peaks. The HO film has a positive remanent polarization (*P*_r_) of 8 μC cm^–2^, with a coercive field (*E*_c_) of 1.5 MV cm^–1^. HZO has a positive *P*_r_ of 9 μC cm^–2^, with an *E*_c_ of 1.2 MV cm^–1^. If the ZrO_2_ layers were purely dielectric, they would have acted as dead layers, reducing the effective electric field across the film. This, in turn, would result in a decrease in remanent polarization and an increase in the coercive field,^19^ which is not observed in this case. Therefore, it can be concluded that HZO and HO samples have effective ferroelectric layers of almost identical thicknesses. It is interesting to note that when multilayers are prepared with ZrO_2_ as the initial layer, poor ferroelectric properties are observed in the multilayer (see the Supporting Information). This is likely due to the nonpolar phase of the initial ZrO_2_ layer. We can speculate that this could be improved if the polar phase would be stabilized in this first layer by optimizing the processing parameters. A similar result is reported by van Gent et al.^20^ in epitaxial rhombohedral Hf_1–*x*_Zr_*x*_–ZrO_2_ superlattices prepared via pulsed laser deposition, indicating that this effect is intrinsic to HfO_2_–ZrO_2_ multilayers, regardless of the fabrication method, type of ferroelectric phase, or microstructure.

**Figure 3:**
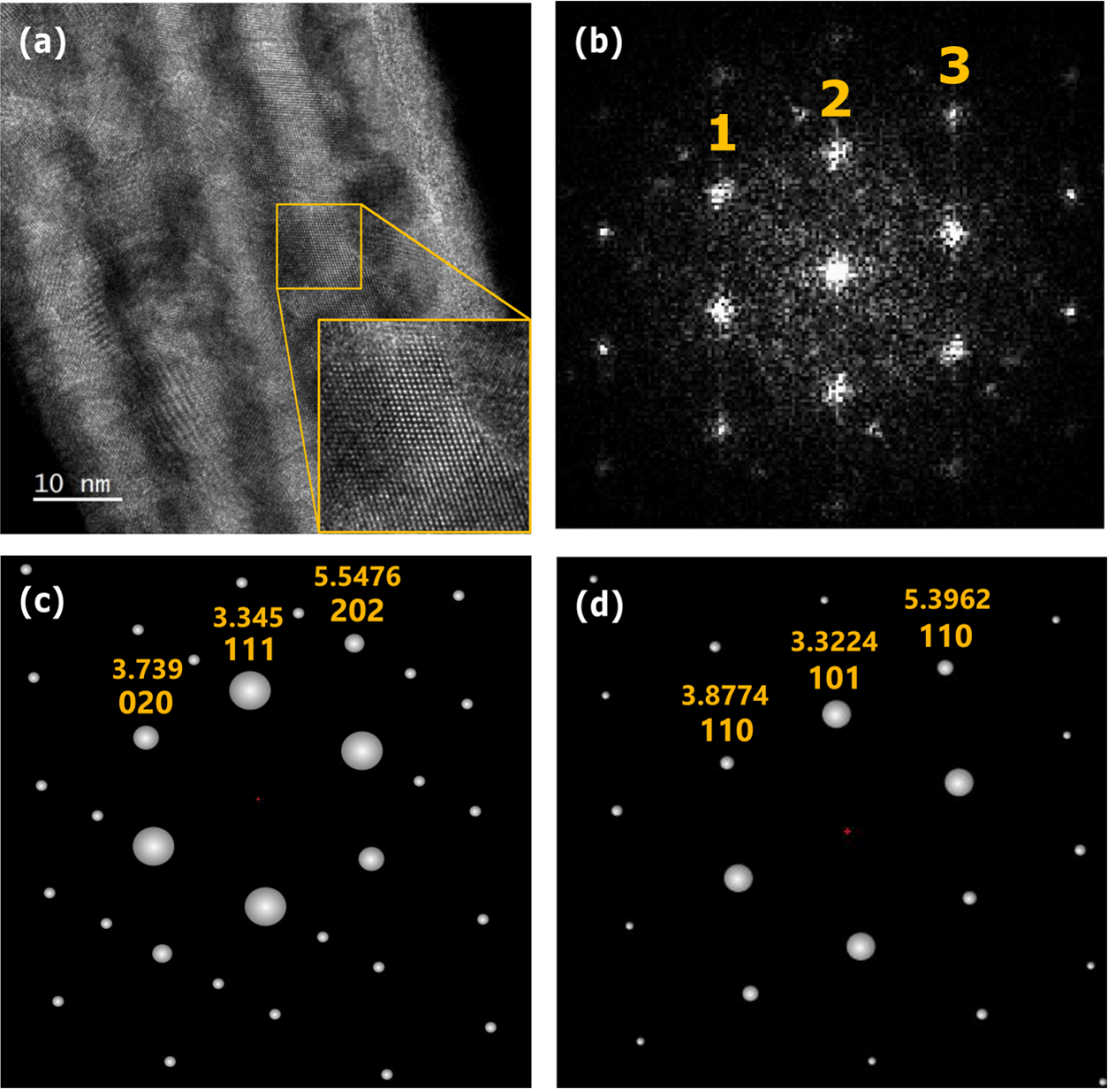
(a) Cross-sectional HRTEM images of the field-cycled HZO multilayer film. (b) FFT diffractogram of the squared area. Simulated diffraction pattern of (c) orthorhombic HfO_2_ along the [1̅01] zone axis (space group *Pca*2_1_) and (d) tetragonal HfO_2_ along the [1̅11] zone axis (space group *P*4_2_/*nmc*).

To gain a better understanding of the phase and grain orientation, cross-sectional high-resolution transmission electron microscopy (HRTEM) was performed on the HZO sample, and the result is shown in Figure 3a. Lattice fringes extending across multiple layers are observed, suggesting a continuation of grains and their orientation through the layers with different compositions. The fast Fourier transform (FFT) diffractogram of an exemplary area is presented in Figure 3b. The FFT experimental pattern was compared with the simulated diffraction patterns from PDF cards of monoclinic (space group *P*2_1_/*c*), orthorhombic (space group *Pca*2_1_), and tetragonal (space group *P*4_2_/*nmc*) phases. It aligns well with the simulated diffraction pattern of the ferroelectric o-phase along the [1̅01] zone axis (Figure 3c) and the nonpolar t-phase along the [1̅11] zone axis (Figure 3d). The ratios of the distances from the center to spots 1 and 2 and those from the center to spots 2 and 3 in experimental FFT are 1.62 and 0.86, respectively. These values are closely aligned with those obtained from simulated patterns of the o-phase and t-phase (see the Supporting Information). The observed ferroelectric switching in the electrical measurements suggests the presence of the o-phase rather than the t-phase in the presented lattice fringe. However, the possibility of the diffractogram corresponding to the m-phase can be ruled out (see the Supporting Information).

**Figure 4:**
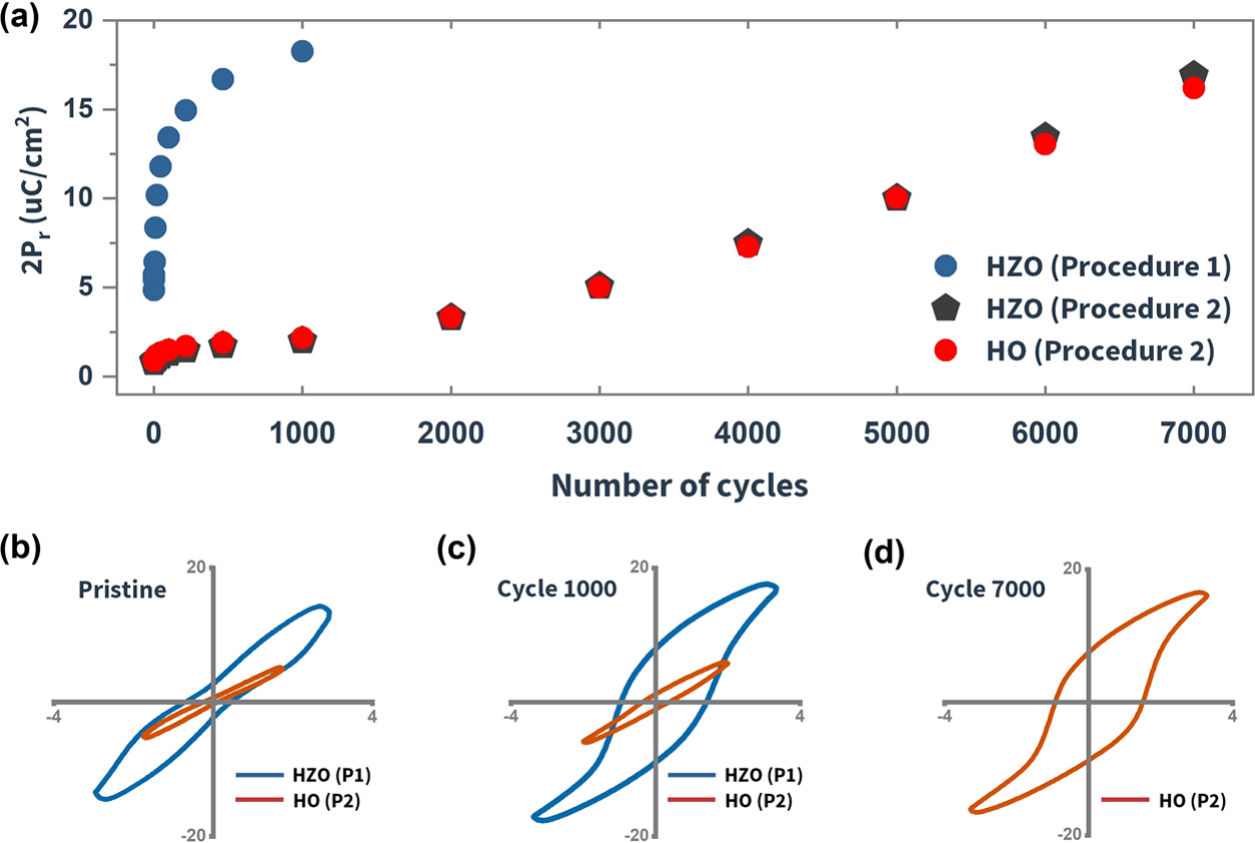
(a) Remanent polarization 2*P*_r_ vs number of wake-up cycles for HZO and HO films. (b) Pristine loops of HZO and HO, acquired from procedures 1 and 2, respectively. (c) HZO and HO loops measured after 1000 cycles following procedures 1 and 2, respectively. (d) HO loop measured after 7000 cycles following procedure 2; for the HZO film, maximum 2*P*_r_ was achieved by procedure 1 (1000 cycles); therefore, no loop is recorded after 7000 cycles [P1: procedure 1, P2: procedure 2].

While the conventional HO and multilayer HZO films show comparable ferroelectric properties, an important difference was observed during wake-up cycling. Figure 4a illustrates the increase of remanent polarization 2*P*_r_ with respect to the number of wake-up cycles. Two distinct sets of wake-up cycles were conducted on the HZO and HO films. The first set, referred to as procedure 1, consisted of a 1000-cycle wake-up at an electric field of approximately 3 MV cm^–1^, exceeding the coercive field of typical HfO_2_ films. The second set, procedure 2, which involved 7000 cycles, was performed as seven consecutive 1000-cycle wake-ups, with the electric field gradually increasing from about 0.8 MV cm^–1^ to around 3 MV cm^–1^. The HO film could not withstand the immediate application of fields exceeding *E*_c_ and typically broke down after approximately 10 cycles, making wake-up cycling using procedure 1 impossible. HZO film, on the other hand, revealed an escalated wake-up behavior upon treating with procedure 1. The pristine loops of HZO (from procedure 1) and HO (from procedure 2) films are presented in Figure 4b, where HZO shows a propeller-shaped hysteresis loop with nonzero remanent polarization, very similar to Hf_*x*_Zr_1–*x*_O_2_ films reported in previous studies.^5,7^ Experimental observations and theoretical modeling have linked that to an electric field-induced phase transition from the tetragonal (T) phase to the orthorhombic o-III phase.^2,21^ Furthermore, studies have shown that propeller-shaped hysteresis loops arise due to defect pinning and are also influenced by the evolution of charged domain walls.^22,23^Figure 4c presents the hysteresis loops after 1000 cycles, showing that HZO reached a maximum 2*P*_r_ of 18 μC cm^–2^. In comparison, the HO film required 7000 cycles to achieve comparable 2*P*_r_ (see Figure 4d). When using procedure 2, HZO showed a wake-up behavior nearly identical to that of the HO sample (see Figure 4a).

Wake-up field cycling is typically performed in hafnia-based ferroelectrics to improve the ferroelectric response through redistribution of defects, typically oxygen vacancies.^24,25^ However, these defects, often located at grain boundaries, interfaces, or domain walls, can also form conductive filaments upon exposure to electric field.^26−28^ Performing wake-up cycles directly at or above the coercive field is therefore detrimental to the dielectric properties of conventional HO films. We hypothesize that the reason multilayer HZO films can endure direct application of high fields is multiple La:HfO_2_–ZrO_2_ interfaces, which disrupt the formation of conductive filaments along the film thickness. Breakdown measurements were performed on both samples (see the Supporting Information), and early breakdown was indeed observed in the HO film. Furthermore, endurance improved from 10^4^ cycles in HO films to 10^5^ cycles in HZO films (see the Supporting Information). Note that enhanced cycling endurance from 10^6^ to 10^9^ cycles is observed in epitaxial rhombohedral Hf_1–*x*_Zr_*x*_–ZrO_2_ superlattices, confirming that multilayering is an effective approach to robust ferroelectric hafnia-based films.^20^ Beyond hafnia, Sun et al.^29^ achieved a breakdown strength of 4.5 MV cm^–1^ in BZT–BCT multilayer films, compared to 1 MV cm^–1^ in single-layer BZT and BCT films of the same thickness. They explained the effect by inhibited propagation and growth of “electric trees” in the films with engineered interfaces. In addition to multilayering, early fatigue in ferroelectric thin films has been reported to be tackled through strategies such as engineering of oxygen vacancies, suppressing defect diffusion, and introducing compositional inhomogeneity.^30−32^

## 4 Conclusions and Outlook

In summary, we demonstrated ferroelectricity in a solution-processed 50 nm-thick HZO multilayer film. The ferroelectric properties of the multilayer thin film were investigated through the characterization of compositional profiles, crystalline phases, and electrical measurements. Although the pure ZrO_2_ films processed were paraelectric in nature, the synthesis of a multilayer structure with HfO_2_ induced a polar phase in ZrO_2_ through the La-doped HfO_2_ layer. The HZO film exhibits ferroelectric properties, with a remanent polarization of 9 μC cm^–2^ and a coercive field of 1.2 MV cm^–1^. Additionally, prepared HZO films can endure higher electric fields due to the presence of multiple interfaces, which significantly reduces the wake-up cycles, an improvement by an order of magnitude compared to the conventional films. This study highlights a novel pathway to enhance the wake-up properties of ferroelectric HfO_2_- and ZrO_2_-based thin films by introducing interfaces, rather than relying solely on solid solutions. This approach can be extended to the growth of thicker multilayer films for piezoelectric applications as the improved wake-up properties and reduced early breakdown during extended cycling help address the challenges typically associated with thicker films.

## Data Availability

The raw data generated in this study have been deposited in the Zenodo repository under https://doi.org/10.5281/zenodo.14893377.

## Supporting Information Available

Polarization hysteresis of the ZHO film, EDS spectrum and FFT diffractogram of the HZO film, and leakage and fatigue measurements of HO and HZO films (PDF)
