# Supplementary material for: Ferroelectric HfO2–ZrO2 Multilayers with Reduced Wake-Up
Source: ACS Omega. 2025 Mar 28;10(13):13141–7. doi: 10.1021/acsomega.4c10603 (PMC11983163; doi:10.1021/acsomega.4c10603)
Supplement: Supplementary file 1 — ao4c10603_si_001.pdf [file ao4c10603_si_001.pdf]

# Supporting information: Ferroelectric $HfO_2$-$ZrO_2$ multilayers with reduced wake-up

Barnik Mandal[*1,3], Adrian-Marie Philippe[2], Nathalie Valle[2], Emmanuel Defay[1,3], Torsten Granzow[1], and Sebastjan Glinsek[1].

[1]Smart Materials Unit, Luxembourg Institute of Science and Technology (LIST), 41 Rue de Brill, L-4422 Belvaux, Luxembourg.

[2]Advanced Analyses and Support Unit, Luxembourg Institute of Science and Technology (LIST), 41 Rue de Brill, L-4422 Belvaux, Luxembourg.

[3]University of Luxembourg, 2 Av. de l'Universite L, L-4365, Esch-sur-Alzette, Luxembourg.

# S1. ZHO film

Figure S1(a) presents a structural schematic of the multilayer ZHO film, which consists of ten 5 nm-thick layers of alternately deposited $ZrO_2$ and La:$HfO_2$, starting with $ZrO_2$ and ending with La:$HfO_2$. Figure S1(b) presents the electrical characterization of the ZHO film. After treatment with procedures 1 and 2, the ZHO film hardly shows switching current.

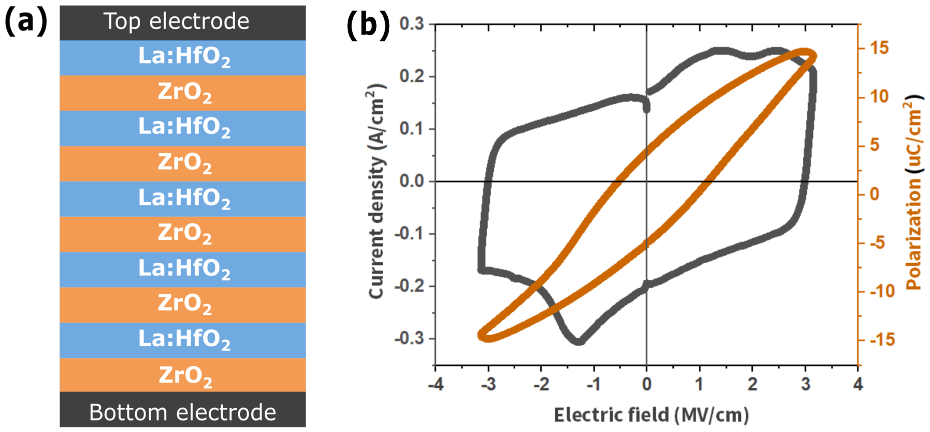


Fig. S1: (a) Structural schematics, and (b) hysteresis loops of the ZHO film after treating with procedure 2.

# S2. Energy-dispersive X-ray spectroscopy (EDS)

The EDS spectrum in Figure S2 was utilized for line profile analysis. The EDS mapping of the cross-section profile of the HZO multilayer film is presented in Figure S3. Note that the green regions observed both at the top and at the bottom of the Hf map (S3 (b)) are an artifact due to the superimposition of the EDS Hf M and Pt M peaks. Similarly, the red regions visible both at the top and at the bottom of the Zr map (S3(c)) is an artifact due to the superimposition of EDS Zr K and Pt M peaks.

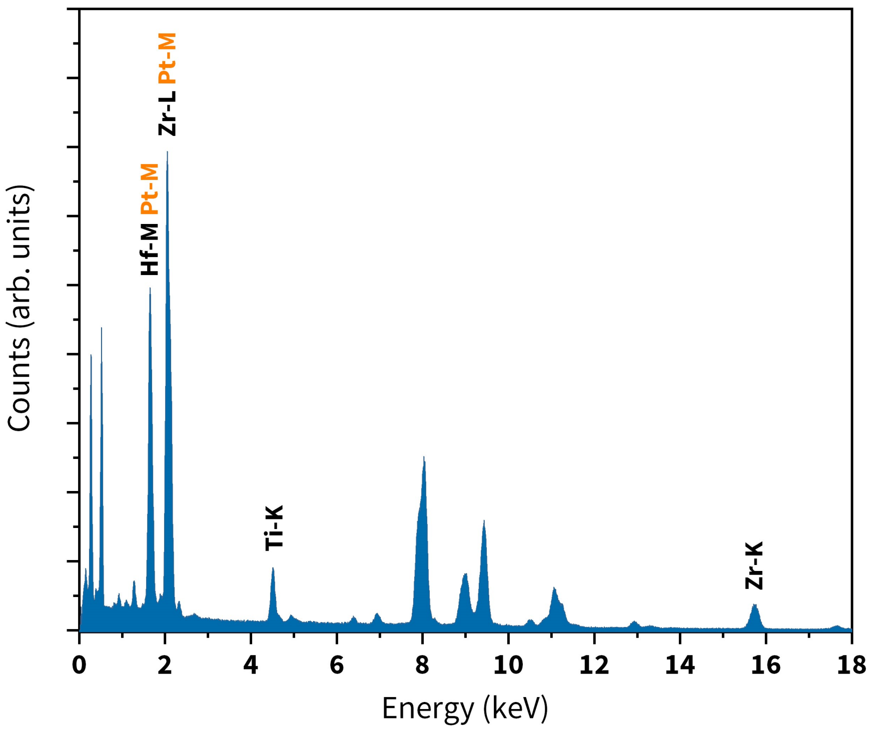


Fig. S2: EDS spectrum of HZO sample cross-section.

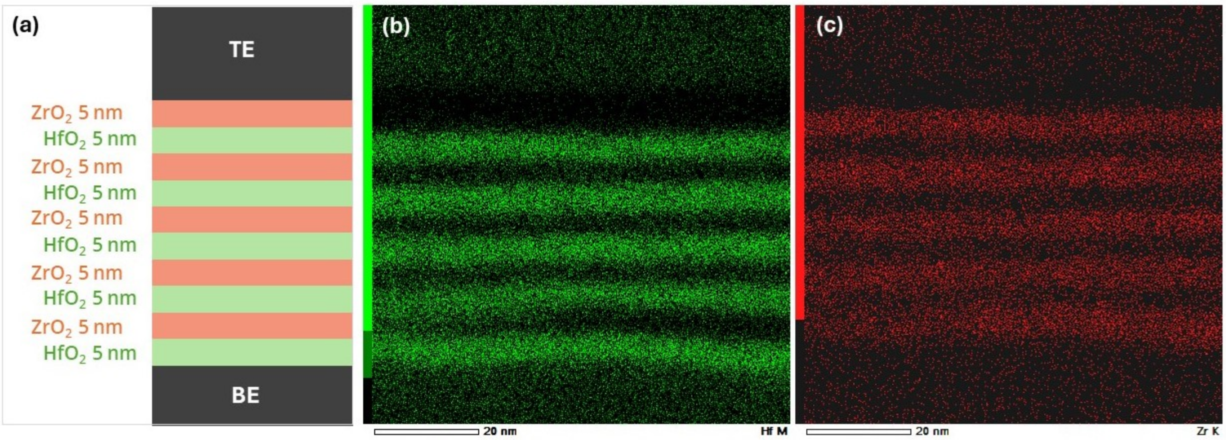


Fig. S3: (a) Multilayer schematic. EDS mapping of the cross-section profile of the HZO multilayer film consisting of (b) hafnia layers extracted from Hf-M and (c) zirconia layers extracted from Zr-K line profiles. TE: top electrode, BE: bottom electrode.

## S3. Fast Fourier Transform (FFT)

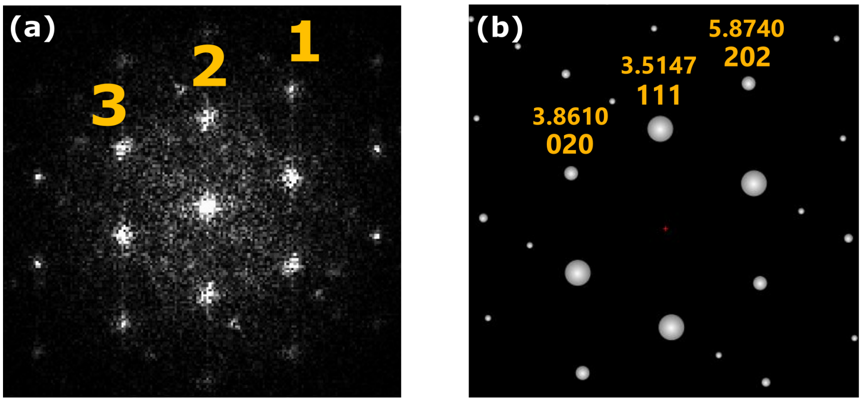


Fig. S4: (a) FFT diffractogram of the sample. (b) Simulated diffraction pattern of monoclinic $HfO_2$ along [-101] zone axis (space group $P2_1c$).

Figure S4 shows the FFT diffractogram of the sample alongside simulated diffractograms for the m-phase. The bright central spot of the bi-dimensionnal FFT is a consequence of the smooth, i.e. long-range, transitions as well as the presence of noise in the original image Figure S4(a). Indeed, the center of the FFT is where the low frequency components of the real space are stored. All other bright spots visible on Figure S4(a) are related to higher frequencies corresponding to short-range periodicities on the original image and their respective spacing are measured from the center of the FFT image. A comparison of the spacings is provided in Table S1. The two-spot ratio of the sample is more closely aligned with the o-phase and t-phase than with the m-phase.

Table S1: Comparison of FFT spot spacing (in $nm^{-1}$) between the HZO sample data and the simulated phases shown in S4.

| | HZO multilayer | o-phase | t-phase | m-phase |
|---|---|---|---|---|
| Spot 1 | 5.29 | 5.54 | 5.39 | 5.87 |
| Spot 2 | 3.25 | 3.34 | 3.32 | 3.51 |
| Spot 3 | 3.77 | 3.73 | 3.87 | 3.86 |
| Ratio (spot 1/ spot 2) | 1.62 | 1.65 | 1.62 | 1.67 |
| Ratio (spot 2/ spot 3) | 0.86 | 0.89 | 0.85 | 0.90 |

## S4. Breakdown and fatigue measurements

Repeated measurements were performed on both the samples. Figure S5 presents the breakdown measurements for one example of each type. The HO films consistently broke down at ∼2.3 MV/cm, while HZO sustained fields up to 4.0 MV/cm and beyond. Leakage current was below the measurement resolution for HO, while the HZO films showed an Ohmic behaviour with a resistivity ranging from $4 \cdot 10^7$ Ωcm to $7 \cdot 10^7$ Ωcm.

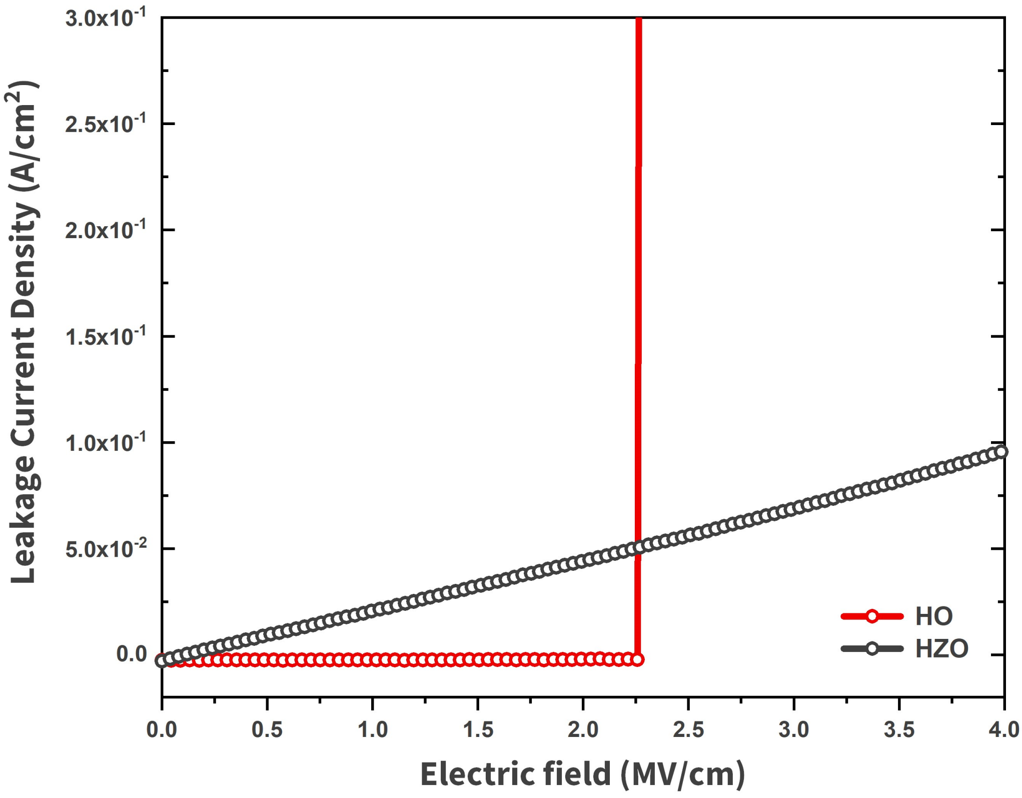


Fig. S5: Breakdown measurements of the pristine HO and HZO films. The measurement was performed on the TF Analyzer 2000 (aixACCT) tool with a step of 200 mV with a waiting time of 100 s for each data point.

Fatigue measurements are shown in Figure S6. The measurements were conducted at 3 kHz with gradually increasing the electric field from 0.8 MV $cm^{-1}$ to 3 MV $cm^{-1}$ (procedure 2). The HO sample experienced breakdown after 7,000 cycles, whereas the HZO multilayer films endured over 100,000 cycles, lasting an order of magnitude longer than the HO film. However, significant leakage current was observed after 50,000 cycles.

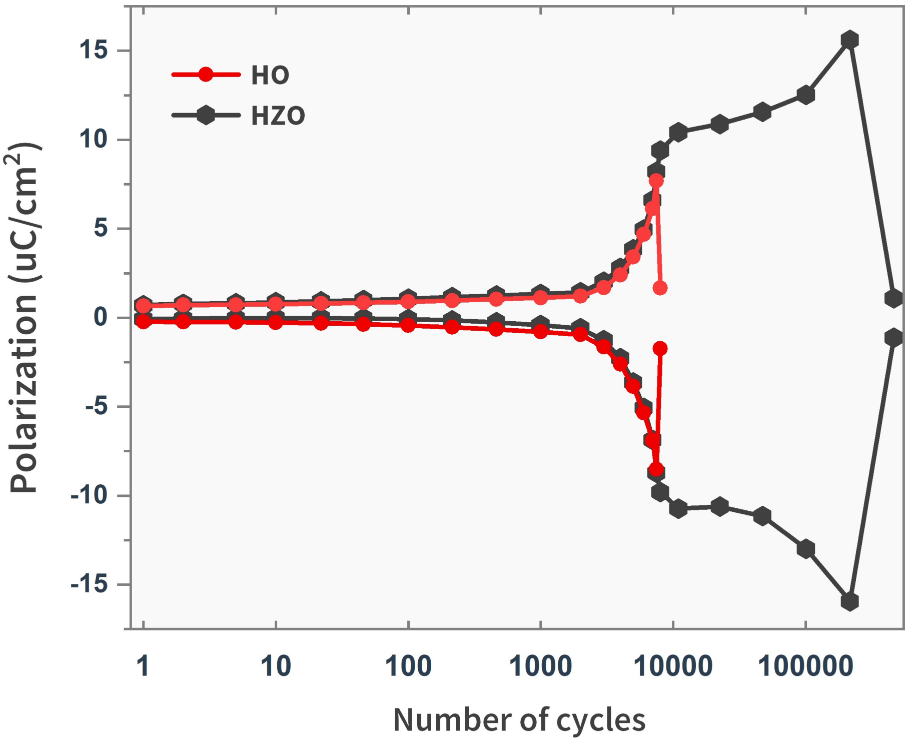


Fig. S6: Fatigue measurements. The measurements were conducted by cycling at 3 kHz with gradually increasing the electric field from 0.8 MV/cm to 3 MV/cm (procedure 2).
